# Application of the CRISPR/Cas9 System to Study Regulation Pathways of the Cellular Immune Response to Influenza Virus

**DOI:** 10.3390/v14020437

**Published:** 2022-02-21

**Authors:** Daria Prokhorova, Natalya Zhukova (Eschenko), Anna Lemza, Mariia Sergeeva, Rinat Amirkhanov, Grigory Stepanov

**Affiliations:** 1 Laboratory of Genome Editing, Institute of Chemical Biology and Fundamental Medicine of the Siberian Branch of the Russian Academy of Sciences, 630090 Novosibirsk, Russia; prohorova1994@gmail.com (D.P.); eschenko96@gmail.com (N.Z.); lemza.ae@yandex.ru (A.L.); mari.v.sergeeva@gmail.com (M.S.); rinatamirkhanov@gmail.com (R.A.); 2 Department of Natural Sciences, Novosibirsk State University, 630090 Novosibirsk, Russia; 3 Laboratory of Vector Vaccines, Smorodintsev Research Institute of Influenza, Ministry of Health of the Russian Federation, 197376 Saint Petersburg, Russia

**Keywords:** CRISPR/Cas9, influenza virus, cellular immune response, cell receptors, RNA processing factors, CRISPR/Cas9 genome screening

## Abstract

Influenza A virus (IAV) causes a respiratory infection that affects millions of people of different age groups and can lead to acute respiratory distress syndrome. Currently, host genes, receptors, and other cellular components critical for IAV replication are actively studied. One of the most convenient and accessible genome-editing tools to facilitate these studies is the CRISPR/Cas9 system. This tool allows for regulating the expression of both viral and host cell genes to enhance or impair viral entry and replication. This review considers the effect of the genome editing system on specific target genes in cells (human and chicken) in terms of subsequent changes in the influenza virus life cycle and the efficiency of virus particle production.

## 1. Introduction

Influenza A virus (IAV) causes acute respiratory infections in humans and remains a continuous and severe threat to public health. Defining and understanding mechanisms for regulating IAV replication is an effective strategy for developing new ways to fight the virus. Currently, there are various strategies for regulating gene-directed replication of influenza virus in mammalian cells, including human cells: utilization of antisense oligonucleotides, application of the RNA interference methods, and clustered regularly interspaced short palindromic repeats (CRISPR)/CRISPR-associated protein (Cas) system.

Antisense oligonucleotides (ASOs) are small-sized single-stranded nucleic acids, which can be employed to modulate gene expression. There are two main mechanisms of ASO action: one is an RNase H-dependent pathway, and the other is a pathway for steric blocking of splicing or translation [1]. Currently, in the fight against viruses such as IAV, the viral RNAs are the main target of ASOs [2,3,4]. On the other hand, regulation of the host-cell genes expression to reduce viral titers can be another target for the application of antisense derivatives [5,6,7]. The main advantages of the ASO strategy are the fast degradation of the target transcript and the simplicity of oligonucleotide design, allowing for rapid gene delivery [8]. However, the use of ASOs is associated with serious problems: high propensity of oligonucleotides to degradation, lack of cell specificity, cytotoxicity, etc. In addition, ASOs can be used to induce transcript degradation, inhibit translation, cause aberrant splicing, or interfere with miRNA maturation [9].

Another way to regulate transcript levels is the RNA interference (RNAi) method. The mechanism of RNAi is realized through the delivery of small synthetic RNAs, such as small interfering RNAs (siRNAs) or short-hairpin RNAs (shRNAs), into cells [10], where they are subsequently incorporated in the RNA-induced silencing complex (RISC). Afterward, the antisense strand of siRNA guides the RISC to the complementary mRNA for cleavage of this target mRNA, leading to specific gene silencing [11]. However, the RNAi method often allows only to reduce the gene expression, resulting in the knockdown but not in the knockout of the selected gene [12]. Properly designed siRNAs have been shown to function as potent inhibitors of influenza virus replication. In mice, the growth of the following IAV strains and subtypes: A/Puerto Rico/8/34 (H1N1), A/Hong Kong/156/97 (H5N1), A/Netherlands/219/03 (H7N7), and A/Hong Kong/1073/99 (Avian H9N2) [13,14]. RNAi method permits the use of diverse cell lines, introduces RNA into cells with high efficiency, and studies the effect of essential genes on phenotypes [15]. Nonetheless, there are major obstacles to the widespread application of the RNAi strategy. The RNAi technology causes temporary effects, incomplete knockdowns, and high off-target effects [16].

**Figure 1:**
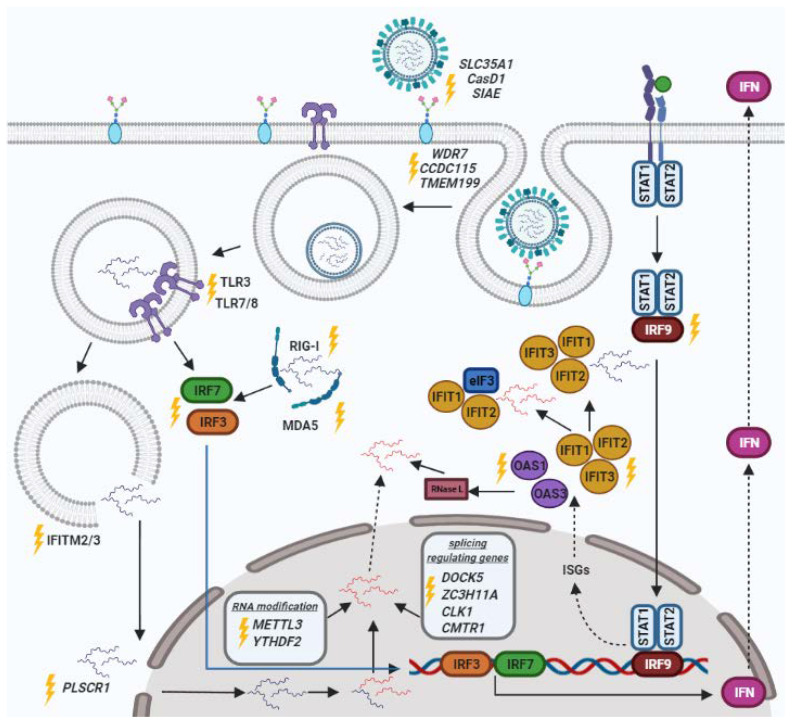
Possible target genes for CRISPR/Cas9 system to adjust the sensitivity of cells to influenza virus. In the figure, the lightning symbol indicates genes whose knockout leads to changes in the virus’s life cycle and affects the susceptibility of cells. RNA marked in red is viral mRNA; RNA marked in blue is viral negative-sense RNA.

The CRISPR/Cas technology seems to be more promising than the other presented strategies. The CRISPR/Cas9 system is a bacterial defense mechanism against phage infection and plasmid transfer in nature [17] that was discovered back in the 1980s, but only in the last 10 years, the technologies have been developed to use this system for genome editing in eukaryotic cells [18]. The CRISPR/Cas9 system includes the Cas9 protein, which acts as a DNA endonuclease; short mature CRISPR RNA (crRNA), which contains the spacer (sequence complementary to the DNA substrate); and trans-activating crRNA (tracrRNA) (sequence complementary to the crRNA) or a chimeric single guide RNA (sgRNA), which combines the crRNA and the tracrRNA [17]. Cas9 endonuclease first recognizes a short conserved sequence-the protospacer-adjacent motif (PAM) [19]. The PAM-proximal 10–12 nucleotides are the seed region of guide RNA that binds complementarily to the target DNA after PAM recognition [20]. Cas9 has two distinct nuclease domains: an HNH-like nuclease domain that cleaves a target strand of DNA substrate and an RuvC-like nuclease domain that splits a non-target strand [21]. Cleavage of the target DNA with Cas9 results in a blunt DNA double-strand break (DSB) at a position three base pairs upstream of the 3’ end of the protospacer sequence, measuring from the PAM [22]. Another example of a CRISPR/Cas system is the CRISPR/Cas13 system. Unlike Cas9, the Cas13 protein has two higher eukaryotes and prokaryotes nucleotide-binding domains (HEPN) and only cleaves RNA molecules [23,24]. In addition, when binding to a complementary target RNA, Cas13 is able to cut not only the target RNA but also any other RNA molecules [25]. The CRISPR/Cas13 system has a low off-target effect and high efficiency [26,27]. The properties of this technology are widely used to create diagnostic platforms, including for the detection of IAV [28,29]. The main gains of CRISPR/Cas9 technology are a higher signal-to-noise ratio, higher reproducibility, and better genome coverage [30]. Moreover, the CRISPR/Cas strategy reduces a gene expression at the level of DNA and RNA. However, there are some disadvantages of using the CRISPR/Cas9 system, such as toxicity, delivery complexity, and off-target effects [30,31]. Unlike RNAi or ASOs methods, the CRISPR/Cas9 system has higher specificity and fewer off-target effects [32]. It helps to increase the understanding of the interaction between the virus and the host cell in terms of both regulatory factors and RNA modifications. Moreover, CRISPR/Cas technology could be aimed at regulating the replication of various respiratory viruses. For example, the CRISPR/Cas13 system is capable of targeting and inhibiting the genome RNA of influenza virus, SARS-CoV-2 RNA fragments, as well as MERS coronaviruses [29,33]. Frieje et al. [29] developed a Cas13-assisted restriction of viral expression and readout (CARVER) platform to detect and cleave the single-stranded RNA viruses such as lymphocytic choriomeningitis virus (LCMV); influenza A virus (IAV); and vesicular stomatitis virus (VSV). This technology can be used clinically to rapidly measure wild-type viral RNA levels and detect specific viral mutations, but it is limited by the toxicity of single-stranded RNA cleavage in eukaryotic cells [29]. Recently, Abbott et al. [33] developed a prophylactic antiviral CRISPR in human cells (PAC-MAN) strategy based on the CRISPR/Cas13 system, which can degrade SARS-CoV2 RNA fragments and live influenza A virus. The authors found that six crRNAs could target more than 90% of all coronaviruses. The PAC-MAN strategy could be used for a possible treatment for COVID-19. However, the further application of this strategy requires additional experiments on the choice of effective in vivo delivery method, evaluation of off-target effects, testing of antiviral efficiency, and specificity in relevant preclinical models [33]. Currently, the most studied CRISPR/Cas system is CRISPR/Cas9, which provides efficient knockout of target genes and allows for investigating the mechanisms of gene regulation during viral infection. Additionally, the CRISPR/Cas9 genome screening technology is the most promising approach to finding targets because this method offers almost unlimited flexibility, fast and efficient targeting of individual genes, and the ability to target multiple genes simultaneously [34]. This review is devoted to the regulation of influenza virus replication due to the “point-like” CRISPR/Cas9-mediated control of gene expression in the host cell. This review considers the genes of the innate immune response, pattern recognition receptors, cell receptors, the genes responsible for the viral penetration into the nucleus, and RNA processing factors as the targets for the regulation of influenza virus replication (Figure 1).

## 2. Genes of the Innate Immune Response

### 2.1. IRF

An important factor that affects the immune response against the influenza virus is interferon (IFN), which helps to eliminate viruses at the early stages of infection. The genes of the interferon regulatory factor (IRF) family play a key role in the regulation of the interferon activation cascade.

In a recent study, Tuerxun et al. showed the IRF family to be involved to varying degrees in response to influenza virus infection [35]. During IAV infection, the largest change was observed in the IRF7 and IRF9 expression levels, which were significantly increased. However, in influenza B virus (IBV) infection, increased IRF2, IRF7, and IRF9 and decreased IRF4 and IRF5 expression levels were found [35].

In work published by Komissarov et al., the CRISPR/Cas9 system was used to knock down the IRF7 gene, and the impact of this gene on the regulation of antiviral response was demonstrated. The IRF7 gene knockout was shown to increase the sensitivity of human HEK293FT cells to IAV [36]. It was also shown that knockout of the IRF7 gene alters the levels of the IFITM (interferon-induced transmembrane) and IFIT (interferon-induced proteins with tetratricopeptide repeats) family genes expression, which play an important role in viral infection inhibition.

In a clinical study [37], it has been demonstrated that among 22 patients with severe influenza infections, one patient had compound heterozygous mutations in IRF7 and that both of her IRF7 alleles fail to upregulate IFN type I and type III. Thus, in case of mutation in both IRF7 alleles, the type I IFN pathway fails to upregulate, leading to an increase in the virus level in fibroblasts and an abolition in type I and III IFNs expression in pDCs (plasmacytoid dendritic cells) [37].

The same pattern is observed for homozygous loss of function IRF9 gene mutations. It has been shown that the IRF9-deficient patient was not exposed to the respiratory syncytial virus (RSV) or human rhinovirus (HRV), which indicates its ability to control various common viruses in vivo, including respiratory viruses other than IAV [38]. As a result of IRF9 deficiency, cells of all types demonstrate an inability to respond fully and effectively to type I IFNs and, by inference, to type III IFNs. The connection between IRF7 and IRF9 deficiency mechanisms suggests the essential role of type I and III IFN responses driven by IRF7 amplification and mediated by IRF9 signaling in influenza virus defense.

Unlike mammals, birds only have IRF7 as a modulator of the type I IFN pathway, missing IRF3. However, they also have other protection systems. In their recent study, Tae Hyun Kim et al. examined the ability of chicken IRF7 to regulate the host response against avian influenza virus (AIV). The authors used a CRISPR/Cas9 system to completely knock out (KO) the IRF7 gene in the DF-1 cell line and performed RNA sequencing on the knocked-out and wild-type cells after H6N2 infection. It was found that the replication rate of AIVs in vitro increased significantly in IRF7^-/-^ cells. Additionally, the effect of IRF7 KO on mTOR (mammalian target of rapamycin) and MAPK (mitogen-activated protein kinase) signaling pathways was studied. The mTOR signaling pathway coordinates metabolic processes, and the MAPK pathway controls a wide range of cellular responses such as proliferation, differentiation, and immune response. The study showed that there was a negative correlation between mTOR and IRF7, whereas p38 MAPK (MAPK13) was upregulated due to IRF7 KO. However, in the case of MHC II family genes, there was a positive correlation to IRF7 expression. Based on the findings, Tae Hyun Kim et al. suggested that IRF7 plays a significant role in the host antiviral response against avian influenza virus (AIV) infection, and the MAPK and mTOR signaling cascades allow modulation of the antiviral response against this virus [39].

### 2.2. IFIT

Previous studies using the siRNA or ASO method suggested that the interferon-induced proteins with tetratricopeptide repeats (IFIT) target the translation and replication suppression of various viruses, including IAV [40,41,42,43,44]. However, using a CRISPR/Cas9 system, Tran et al. [45] have shown that IFIT2 promotes the gene expression of the influenza virus. Moreover, due to the binding of IFIT2 to viral mRNAs, IAV replication is enhanced. In addition, the authors have demonstrated that IFIT3, like IFIT2, has proviral activity and is able to regulate the expression of viral proteins during IAV infection.

### 2.3. IFITM

The interferon-induced transmembrane (IFITM) family consists of a number of proteins located in cytoplasmic and endosomal membranes [46]. One of the main functions of these proteins is to inhibit the release of the viral genome from endosomes into the cytosol [47,48].

The influence of the IFITM family genes on influenza virus infection has been established in vitro using the CRISPR/Cas9 system [49]. The infection of HeLa cells with influenza virus showed that a decrease in the expression of IFITM2 or IFITM3 increased the cell susceptibility to the virus by about two or four times, respectively, compared with control cells. At the same time, HeLa clones with a complete knockout of both IFITM2 and IFITM3 or the three isoforms simultaneously demonstrated the increase in the infection level by more than 20 times. No significant difference in influenza virus infection of IFITM2/3 knockout clones or clones lacking all three genes (IFITM1-3) was observed in both intact and IFN-α treated cells, suggesting that IFITM1 does not play a significant role in the prevention of IAV infection. Moreover, it has been demonstrated that increasing the amount of IFITM3 protein in IFITM2/3 knockout cells reduces the infection of cells with the influenza virus, depending on the level of the added IFITM3 protein. In addition, a decrease in infection was also observed when the level of IFITM2 was restored. Thus, the authors have found that IFITM3 acts in a dose-dependent manner to restrict a broad spectrum of viruses [49]. Earlier, in the study by Bailey and colleagues, it was shown that the IAV susceptibility in genetically modified mice lacking genes equivalent to human IFITM family members was increased [48]. A similar effect was obtained for mice lacking only IFITM3. These results demonstrate that within the IFITM family, IFITM3 plays the predominant role in the regulation of IAV infection.

The ability of the IFITM family members to limit influenza infection has also been demonstrated in a mouse model. Infected mice lacking IFITM3 displayed fulminant viral pneumonia due to uncontrolled replication of the virus in cells. A similar effect has been confirmed in vitro. It has been shown that antiviral functions are restored after the addition of the IFITM3 protein. The involvement of the IFITM3 protein in influenza virus infection has also been confirmed in humans. It was found that a statistically significant number of hospitalized individuals have the C allele of the rs12252 single nucleotide polymorphism (SNP rs12252-C) that alters a splice acceptor site [50].

## 3. Pattern Recognition Receptors (PRRs)

During influenza infection, the innate immune system recognizes viral antigens using a range of pattern recognition receptors (PRRs) such as Toll-like receptors (TLRs) and RIG-like receptors (RLRs). The RLR family is composed of three cytoplasmic RNA-recognizing proteins: LGP2 (Laboratory of Genetics and Physiology 2), MDA5 (Melanoma Differentiation-Associated protein 5), and RIG-I (Retinoic acid-inducible gene I). It is also known that RIG-I and MDA-5 are involved in the activation of interferon in response to the influenza virus [51].

### 3.1. TLR

Toll-like receptors (TLRs) recognize a large number of structurally conserved molecules derived from bacteria and viruses and activate cellular immune responses. Moreover, TLR3 and TLR7/8, both located in endolysosomes, recognize double-stranded RNA (dsRNA) and single-stranded RNA (ssRNA) ligands, such as influenza virus and others [52]. Han and colleagues created TLR7, TLR8, and TLR3 knockout human-induced pluripotent stem cell lines (hiPSCs) using the CRISPR/Cas9 system and characterized the morphology and karyotype of these cells, the level of corresponding knockout TLR protein, and differentiation potential of these cell lines in vivo. The obtained knockout cell lines are promising models for further study of the specific role of TLRs in various viral infections [53,54,55]. Lee and colleagues performed siRNA-mediated gene silencing of cMDA5, cTLR3, and cTLR7 as well as CRISPR/Cas9-mediated individual and double knockout of cMDA5 and cTLR3 in chicken embryonic fibroblast DF-1 cells to determine their roles in the IFN-mediated innate immunity in response to avian influenza virus infection. It was shown that the activity of the IFN-β promoter fell by about 10-fold, 3-fold, or 1.8-fold after infection with AIV if cMDA5, cTLR3, or cTLR7, respectively, was silenced. These results suggested that cTLR7 was neither involved in IFN-β signaling in response to AIV in chicken compared to cMDA5 and cTLR3 nor played a significant role in sensing RNA ligands. In addition, as a result of studying knockout lines, the authors found that cMDA5 played a pivotal role in the capture of RNA ligands in DF-1 cells while cTLR3 played only a complementary role. It is important to note that the AIV titer in the clone with the cMDA5 knockout was shown to be about 30-fold higher than that in intact DF-1 cells [56].

### 3.2. MDA-5

MDA-5 is a member of the evolutionary conserved RIG-like helicase family of PRRs (pattern recognition receptors). To date, RIG-I is thought to be the chief booster of the immune response during influenza infection in mammals. Since RIG-I is absent in chicken and ChMda5 (chicken Mda5) knockdown does not appear to affect influenza proliferation, it is assumed that ChMDA5 could compensate for this function [57]. However, the knockout of this gene using the CRISPR/Cas9 system resulted in significant growth of AIV in cMDA5-lacking cells. These data indicate that cells become more permissive to virus growth due to reduced IFN-mediated antiviral activity and because of the impaired production of IFN-β in particular [56].

### 3.3. RIG-I

The role of RIG-I in the regulation of the immune response to the influenza virus was confirmed in a mouse model [58]. RIG-I-KO mice, which were generated by homologous recombination of ES cells [59], showed failure in the production of IFN type I and the subsequent activation of the adaptive immune response (T-cell responses) against influenza viruses [58].

The study conducted by Thulasi Raman et al. has identified a RIG-I analog located in the nucleus. The nuclear arrangement of the RIG-I protein makes it significant for the restriction of nuclear replicating viruses, such as the influenza virus [60]. Recently, the RIG-I-dependent signaling pathway was explored by Yap et al. using the CRISPR/Cas9 mediated knockout of RIG-I and ANXA1 [61]. Knockout of ANXA1 in A549 cells led to a decrease in the expression of interferon-stimulated genes, such as IFIT1 upon IAV infection and a decrease in the expression of RIG-I basally and post-infection. At the same time, the RIG-I knockout cells demonstrated the basal level of ANXA1 compared to control A549 cells. For both knockout cell lines, no change in cell viability was observed upon IAV infection, while infected control cells exhibited a significant reduction in cell viability. The authors concluded that ANXA1 plays a role in RIG-I-mediated IAV-induced apoptotic cell death [61].

## 4. Cell Receptors

### 4.1. OAS Family

Proteins of the OAS family (2′-5′ oligoadenylate synthase) constitute another mediating factor responsible for the activation of interferon in response to influenza virus and induce the activation of ribonuclease L (RNase L), leading to the degradation of viral RNAs [62].

In a recent study [63], the authors knocked out the OAS1 and OAS3 genes in a human leukemia monocytic cell line and HT-1080 fibrosarcoma cell line using the CRISPR/Cas9 system. OAS1 and OAS3 were first shown to inhibit the expression of chemokines and interferon-responsive genes in response to viral infection. However, the stimulations of the two OAS proteins yielded different results, indicating that the TLR3 and TLR4 receptors served as stimulation for OAS1, while RIG-I and MDA5 stimulated OAS3. These data demonstrated the OAS-dependent regulation of innate immune signaling in human macrophages, suggesting wide possibilities for viral infection treatment [63]. Knockout of the OAS family genes in the A549 cell line by the CRISPR/Cas9 system increased the viral replication as compared with control cells. Additionally, inhibition of the OAS3 gene caused the highest increase in influenza virus titer, which suggests that OAS3 is the main OAS isoform responsible for the activation of RNase L [64].

### 4.2. Sialic Acids

Sialic acids (Sia) are nine-carbon monosaccharides typically found in invertebrates. They are commonly presented as terminal residues of cell surface glycans and are known to underlie diverse biological events ranging from cell adhesion to immunity and inflammation. It was demonstrated earlier that IAV, IBV, ICV, IDV, and enterovirus D68 use sialic acids to penetrate cells [65]. To identify the main host factors affecting the replication of IAV (avian strain H5N1), Han et al. have knocked out genes involved in the biosynthesis of sialic acids throughout the genome (GeCKO screening) in human lung epithelial cells (A549) [66]. This method has shown that the SLC35A1 sialic transporter is an important factor for the penetration of IAV into cells and that capicua protein (CIC) is a major negative modulator of cell-mediated immunity.

Another actively studied feature of sialic acids is their O-acetyl modifications. The effect of Sia-O-acetyl modifications on IAV, IBV, ICV, and IDV infection has been investigated in various human and canine cells [67]. Using CRISPR/Cas9 technology, the sialate O-acetyltransferase gene (CasD1) was knocked out or overexpressed, and the sialate O-acetyl esterase gene (SIAE) was knocked out. Modulations in CasD1 and SIAE expression revealed that these genes partially regulate the level of O-acetyl modifications; however, there are other ways of regulation. Low levels of O-acetyl modifications of sialic acids are obstructive for ICV or IDV to penetrate the cell, whereas they had no obvious effect on IAV and IBV infection.

## 5. Genes Responsible for Viral Penetration into the Nucleus

The study by Luo et al. has shown the PLSCR1 (Phospholipid Scramblase 1) protein to be an interacting partner of the influenza nucleocapsid protein (NP) in mammalian cells. This interaction is important for the regulation of IAV replication, as siRNA knockdown or CRISPR/Cas9-mediated knockout of PLSCR1 expression increases virus replication. It was found that the inhibitory effect of PLSCR1 involves the formation of its complex with the viral NP and importin α, which prevents the incorporation of importin β into the complex, impairs the nuclear import of NP, and suppresses the virus [68].

## 6. RNA Processing Factors

### 6.1. Splicing Regulating Genes

Splicing factors are essential for influenza virus replication. IAV uses alternative splicing to produce several of its proteins. The functional roles of the dedicator of cytokinesis 5 (DOCK5) protein [69] and CLK1 protein (CDC Like Kinase 1) in the influenza virus life cycle were studied using the CRISPR/Cas9 genome editing system [70].

Dock5 protein is a large polypeptide implicated in an intracellular signaling network and located in the plasma membrane to stimulate cell proliferation and migration [71]. To elucidate the role of DOCK5 in the replication of influenza A (H1N1 and H3N2) viruses, this gene was knocked out in A549 human lung epithelial cell lines using the CRISPR/Cas9 system. The knockout led to a decrease in the titer of the influenza virus. It has been shown that DOCK5 is a host factor capable of regulating the traffic, replication, and splicing of influenza virus genes in the cell. Another possible function of this protein may be to suppress host defense responses [69].

CLK1 is a human protein kinase that plays the role of essential host factor in IAV replication and can affect the splicing of viral mRNA. CLK1 gene knockdown in A549 cells resulted in reduced IAV replication and decreased virus titer. This effect was observed in both A549 human cells and mouse models. Moreover, it was shown that among the CLK family kinases, only the CLK1 isoform is required for the successful replication of IAV in vitro and in vivo [70].

### 6.2. Genes of RNA Modification

RNA modifications are another factor affecting the life cycle of IAV. Courtney’s research (2017) is the main study of the effect of RNA modifications on IAV replication and pathogenicity [72]. In this work, the effect of an N6-methyladenosine (m6A) modification on the expression and replication of the influenza A genome has been examined. The expression of the methyltransferase METTL3 gene, which is responsible for introducing m6A modifications, was blocked by the CRISPR/Cas9-mediated knockout in the A549 human lung epithelial cell line. METTL3 knockout was found to cause a decrease in viral structural protein levels as well as in virus titer and viral mRNA levels. In contrast, overexpression of YTHDF2 (YTH domain family 2) induced a significant increase in virion production in A549 cells, but this effect was not observed with overexpression of YTHDF1 or YTHDF3. Thus, the study conducted by Courtney et al. has provided the first evidence that m6A RNA modifications upregulate the replication of IAV; however, the underlying mechanism of the effect has not been presented and justified.

Later Cortney’s data were confirmed in a study aimed at exploring the mechanism of m6A control via modifying the innate immune response to infection. In their work, Winkler et al. (2019) described the pathway of m6A-mediated destabilization of interferon β (INFβ), which can affect the propagation of viruses [73]. IAV infection of cells with the CRISPR/Cas 9 mediated knockout of METTL3 or YTHDF2 was accompanied by an increase in the IFNβ level, a higher expression of interferon-stimulated genes, and inhibition of the expression of IAV genes. Thus, the CRISPR/Cas9 technology aimed at the path of RNA modification allows the regulation of the influenza virus replication. Further, the use of the CRISPR/Cas system would help to understand the mechanism of the effect of RNA modifications, including m6A, on the expression of viral genes.

## 7. CRISPR/Cas9 Genome Screening

Now the CRISPR/Cas9 screening technology is being actively developed and applied in many scientific studies. Genome-wide screening clarifies which specific gene should be knocked out to increase or suppress viral replication in each case.

One such study is the research by Heaton et al., in which the authors performed a CRISPR/dCas9 genome-wide overexpression screening on the cell line A549 to identify the host factors that block the IAV infection. They found that the B4GALNT2 (Beta-1,4-N-Acetyl-Galactosaminyltransferase 2) gene, which encodes glycosyltransferase and is not usually expressed in the lungs, can inhibit various strains of AIV. In addition, B4GALNT2 overexpression caused a significant reduction in both initial virus infection and expansion. Thus, altering B4GALNT2 expression is a promising way to completely prevent IAV infection [74].

In work [75] by Karakus et al., a CRISPR/Cas9 genome-wide screen has been used to study the genes involved in the entry of novel bat IAVs (H17N10 and H18N11). The combination of CRISPR/Cas9 technology and transcriptomic profiling of susceptible versus non-susceptible cells revealed that the major histocompatibility complex class II (MHC-II) human leukocyte antigen DR isotype (HLA-DR) is an important entry mediator for bat IAVs. In addition, the authors have generated MHC-II-deficient mice and have confirmed that these mice are resistant to bat IAV infection.

Another study [76], using CRISPR/Cas9 genome-wide screening, has identified that underexplored host factors WDR7, CCDC115, and TMEM199 are necessary for influenza virus entry and are not essential for cell viability. These genes are shown to be important during the early stages of the IAV infection, and their knockout reduces viral infection. In addition, a human mRNA cap methyltransferase CMTR1 has been revealed as a new host dependency factor of IAV. The knockout of this gene increases the IFIT gene family expression in infected cells and inhibits viral replication [76].

In the research [77], the authors have presented a novel pooled genome-wide CRISPR/Cas9 screening strategy that uses a replication-defective reporter virus and Fluorescence Assisted Cell Sorting (FACS). This technology was applied to identify restriction factors in a given vaccine production cell line as a method validation for the potential implementation in generating high viral yields in cell-based vaccine production systems. Using the HEK-293SF cell line, the authors have discovered 64 putative influenza restriction factors, including RAE1, NUPL2, TSC1, DDX6, DPC2, SMG9, and UPF2. However, a relatively minor impact on virus replication has been found for well-known factors, such as interferon and RIG-I. In addition, the authors suggest that the host cell metabolic state plays a key role in the maintenance of influenza virus reproduction.

In a recent study [78], using a genome-wide CRISPR/Cas9 gene knockout screen, Song et al. have identified a novel host factor—the single-pass type I transmembrane protein immunoglobulin superfamily DCC subclass member 4 (IGDCC4) that facilitates the endocytosis of lethal H5N1 influenza virus infection. The authors have performed in vitro and in vivo studies and have shown that IGDCC4 reduced the replication of the virus in A549 cells, and IGDCC4-knockout mice increased resistance to H5N1 virus infection. However, they have specified that the cellular protein IGDCC4 does not involve in the initial attachment of the influenza virus to the cell surface. Moreover, the authors suggest that influenza virus attachment and endocytosis may be mediated by different cellular proteins.

The experimental application of CRISPR/Cas9 screening generates some issues worth mentioning. One of them is the presence of some false-positive or false-negative results that are difficult to analyze and interpret. Moreover, the results of various screenings are often not in agreement with each other. Therefore, the gene sets of several previous screenings were compared in the supplementary information of a study by Sharon et al. The comparison of the screenings had practically no overlapping genes. This confirms that the results obtained with the CRISPR/Cas9 screening approach depend on the experimental conditions, including the type of genomic library, the virus strain used to infect cells, the type of cell line in which the experiment is carried out, and others.

## 8. The Applications of CRISPR/Cas9 System

At the present time, the CRISPR/Cas9 system is the most promising and widespread technology for detecting viruses, including IAV [79] and studying their assembly [80,81,82], budding [83], release [84], and spread [85].In a study [85], using a CRISPR/Cas9-mediated knockout technique, the authors have generated the tumor protein 53 (p53) null A549 cells to explore the susceptibility of these cells to IAV infection. They showed that p53null cells significantly reduced the viral spread when infected with influenza A compared with control cells [85]. Another research group demonstrated that tumor susceptibility 101 (Tsg101) gene-deficient A549 cells, created by the Cas9/CRISPR method, were resistant to IAV infection [84]. The authors suggested that Tsg101 plays a critical role in the transport of HA on the cell surface prior to the release of IAV [84]. In a study [83], Zhao et al. have investigated the relationships of IAV and two genes: cytidine monophosphate N-acetylneuraminic acid synthetase (CMAS) and ST3 beta-galactoside alpha-2,3-sialyltransferase 4 (ST3GAL4). Using the CRISPR/Cas9, they created knockouts of these genes in newborn pig tracheal epithelial (NPTr) cells. The results showed that the loss of both genes significantly inhibits IAV adsorption, and CMAS and ST3GAL4 are required for IAV attachment and entry [83]. In addition, the CRISPR/Cas9 method can be used to study IAV assembly. For example, in the work by Han et al., it was found that the Rab11 is important for influenza virus genome assembly and production of infectious virus particles [80].

Another promising direction for the application of the CRISPR/Cas9 system is the creation of DNA base editors and prime editors. The advantages of these technologies compared to the original CRISPR/Cas9 system are decreasing cytotoxic effects, low off-target effect, and no serious long-term immune complications [86,87]. Both gene tools have great therapeutic potential, so base editing technologies have shown promising preclinical results in devastating genetic disorders such as Hutchinson–Gilford progeria [86,88].

Moreover, the CRISPR/Cas systems can be used as tools for antiviral therapy [89] and for identifying drug on- and off-targets [90].

## 9. Conclusions

A major problem in the fight against IAV is the steady evolution of surface antigens in response to force from the host’s immune system. Identification of host factors required for IAV reproduction and study of IAV regulation mechanisms will permit to suppress the viral replication at different stages of the viral life cycle.

The CRISPR/Cas9 screening shows limited sensitivity but excellent specificity in detecting host factors that act very early in viral replication. Another important property of this approach is multiplexing (targeting multiple loci), which allows combating the possible evolution of viruses and permits to simultaneously inhibit and overexpress the distinct genetic targets [91]. However, the presence of the off-target effects limits the application of the CRISPR/Cas9 system [32]. Currently, the approaches of reducing the off-target activity of the system are being widely developed. One of the main methods is the introduction of modifications into guide RNAs [92,93], the creation of mutant Cas proteins [94,95], and the finding of new methods of genomic editing based on the CRISPR/Cas9 system [96,97].

The CRISPR/Cas9 system provides a point knockout of genes in the host cell, thereby suppressing or enhancing viral replication at different stages of the viral life cycle. Studies using this approach show the possibility of promoting viral infection. However, a single gene point knockout does not cause the effect necessary to significantly change the virus viability. Creating a cell line with a simultaneous knockout of multiple targets could be a possible solution to this issue, provided that these targets are not involved in any vital processes in human cells. The genes considered in this review can act as the potential targets: IRF, IFIT, IFITM, TLR, MDA-5, RIG-I, OAS family, genes of sialic acids synthesis, PLSCR1 gene, splicing regulating genes, and genes of RNA modification.
